# Retraction: Green synthesis, characterizations of silver nanoparticles using sumac (*Rhus coriaria L.*) plant extract and their antimicrobial and DNA damage protective effects

**DOI:** 10.3389/fchem.2026.1855585

**Published:** 2026-04-27

**Authors:** Frontiers Editorial Office

The journal retracts the 25 August 2022 article cited above.

Following publication, the publisher found evidence of an undisclosed conflict of interest that undermined the integrity of the peer review process. An investigation, which was conducted in line with Frontiers policies, found concerns with the scientific integrity of the article. As the scientific integrity of the article cannot be guaranteed, and in adherence to the recommendations of the Committee on Publication Ethics (COPE), the article is retracted.

This retraction was approved by the Chief Executive Editor of Frontiers. The authors received a communication regarding the retraction and had a chance to respond. This communication has been recorded by the publisher.

